# Semantic Negative Priming From an Ignored Single-Prime Depends Critically on Prime-Mask Inter-Stimulus Interval and Working Memory Capacity

**DOI:** 10.3389/fpsyg.2020.01227

**Published:** 2020-06-09

**Authors:** Montserrat Megías, Juan J. Ortells, Carmen Noguera, Isabel Carmona, Paloma Marí-Beffa

**Affiliations:** ^1^Department of Psychology, University of Almería, Almería, Spain; ^2^CEINSA, Health Research Centre, University of Almería, Almería, Spain; ^3^School of Psychology, University of Wales Bangor, Bangor, United Kingdom

**Keywords:** working memory capacity, individual differences, negative priming, attentional control, masking pattern

## Abstract

The aim of this study is to examine the link between working memory capacity and the ability to exert cognitive control. Here, participants with either high or low working memory capacity (WMC) performed a semantic negative priming (NP) task as a measure of cognitive control. They were required to ignore a single prime word followed by a pattern mask appearing immediately or after a delay. The prime could be semantically related or unrelated to an upcoming target word where a forced-choice categorization was required. Each type of mask (immediate vs. delayed) appeared randomly from trial to trial. Results demonstrated that, when the ignored prime was immediately followed by the mask, neither of the groups (high or low WMC) showed reliable NP. In clear contrast, when the mask onset was delayed responses latencies were reliably slower for semantically related trials than for unrelated trials (semantic NP), but only for the high WMC group. The present results clearly demonstrate that semantic NP from single ignored primes depends on both the masking pattern that follows the prime (immediate vs. delayed mask), and on working memory capacity.

## Introduction

Responding to a probe target stimulus can be slower and/or less accurate if it appeared as an ignored prime distractor in a preceding trial (Tipper, 1985). This effect has been named negative priming (NP) and is usually found in selective attention tasks where both prime and probe displays present the target stimuli accompanied by concurrent distractors. NP has been demonstrated across a wide range of stimuli and task demands (Fox, 1995; Tipper, 2001; Frings et al., 2015). NP is generally reported when the distractor is de-selected against in defiance of a simultaneous target. Nevertheless, further research has successfully produced this effect even when the prime display contains a single ignored stimulus in an otherwise empty visual field (i.e., single NP; Milliken et al., 1998; Ortells et al., 2003, 2016b; Frings and Wentura, 2005; Noguera et al., 2007; Chao and Yeh, 2008).

An influential explanation of the NP effect is in terms of an inhibitory attentional mechanism that results from actively ignoring irrelevant information (Tipper, 1985; Tipper and Cranston, 1985; Houghton and Tipper, 1994). The inhibitory account of NP conceives selection as a dual process in which an excitatory mechanism acting to enhance target information works together with an inhibitory mechanism acting to suppress distraction. Residual inhibition associated with previously ignored information produces a response delay when later appears as a target (for other accounts see Neill et al., 1992; Milliken et al., 1998; Tipper, 2001).

Irrespective of how NP is explained, there seems to be increasing evidence that the effect relies on the availability of control resources (Engle et al., 1995; Lavie et al., 2004; De Fockert et al., 2010; Ortells et al., 2016a). A common way to study this relationship is by evaluating the processing of distractors while varying mental load in a concurrent working memory (WM) task. Thus, several studies have demonstrated that an ignored prime distractor produces reliable NP only when the concurrent memory task demands are low. When these demands are high, however, the NP effect can disappear or be reversed to positive priming (Engle et al., 1995; Chao and Yeh, 2008; De Fockert et al., 2010; Chao, 2011).

Converging evidence comes from research studying correlations between working memory capacity (WMC) and NP. In these studies, researchers typically study differences in NP across groups of participants with extreme high and low WMC (i.e., scoring in the upper vs. lower quartiles in complex span WM tasks). Results have revealed reliable individual differences in showing NP effects as a function of WMC such that only participants with high WMC showed NP, while low WMC did not (Conway et al., 1999; Long and Prat, 2002).

An additional source of support comes from studying NP in populations believed to have reduced WMC, as it is the case of older people (Mayas et al., 2012). Relative to younger adults, their older counterpart display the predicted reduced NP from irrelevant distractors, usually paired with increased interference in conflict tasks (e.g., Stroop). This pattern of high interference and low NP is understood to reflect difficulties suppressing irrelevant information (Mayas et al., 2012). In this sense, having low WMC may have a similar effect on NP as performing a concurrent task demanding a high WM load.

It should be noted that most of prior work exploring the reliance of NP on control resources has employed a repetition (or *identity*) NP procedure in which the prime stimulus is *repeated* as the target on the following probe display. Recently Ortells et al. (2016b) have demonstrated that individual differences in WMC also modulate NP at a semantic level of representation. In that study, two groups of participants with low and high WMC performed a lexical decision (word/non-word) on a probe central target that was preceded by a single prime word that appeared for a very short time (50 ms) on an otherwise empty visual field. The prime word was highly associated to the target (e.g., *tiger*-*lion*) on 50% of word trials, whereas it was unrelated to the target on the remaining 50% of word trials (e.g., *tiger-face*). Participants received instructions to attend to or to ignore the prime word, with attention instructions varying randomly from trial to trial. If the prime was preceded by YES (in green), participants should “attend to and remember” that stimulus, as it would further be tested in a memory task. Conversely, if it was a NO (in red), then they should ignore and treat it as a distractor, which would disrupt their memory of the targets (Noguera et al., 2007, Exp. 4). Results showed that the attended primes produced a positive priming (PP) effect for participants with low and high WMC, but the semantic priming from the ignored words changed with WMC. Semantic NP appeared only in the high WMC group, turning into PP for the low WMC participants.

A peculiar aspect of the semantic NP task used in Ortells et al. (2016a) is that participants had to change their mental set about how to process the single prime word in a random way according to a preceding cue. Therefore, they had to attend (and remember) to the single prime in half of the trials, whereas they were required to ignore it on the remaining trials (Ortells et al., 2003; Noguera et al., 2007). In addition, the complex span tasks often used to assess WMC also required continuous task switches (Unsworth et al., 2005, 2009). When performing these complex span tasks, participants have to alternate and change their attention between retaining in memory a series of items (e.g., letters; spatial locations) of variable length, and manipulating a different type of information in a concurrent task (e.g., verbal -number calculations- or visuospatial-symmetry). It is unclear whether WMC affects inhibitory processes directly or the ability to activate different task goals in a sustained way.

### Current Study

As explained previously, it remains unclear whether the changes in priming pattern from single primes according to WMC can also be found with a NP task that does not require task switching. A first aim of this study is to determine whether the reported dependence of semantic NP on WMC could also be found in a NP task in which participants have to ignore the single prime on every trial.

To this end, we used a semantic NP task similar to that previously used by Daza et al. (2007) in which participants had to make a forced-choice categorization judgment (animal vs. body part) on a single target word. The target was preceded (600 ms before) by a single 33-ms prime word, which participants were encouraged to actively ignore on every priming trial. As in the study by Daza et al. (2007), (Ortells et al., 2016b), the primes and targets were strongly closely associated words of the same semantic category on 50% of trials (related) and they belonged to different semantic categories on remaining trials (unrelated). The ignored prime word was followed either immediately or after a delay by a pattern mask, with the type of masking (immediate vs. delayed) being manipulated within participant and presented in a random way (Daza et al., 2007). On half of trials (delayed masking condition), the prime word was followed by a 314-ms blank screen, and then by a 253-ms pattern mask presented until the appearance of the target display. Therefore, the inter-stimulus interval –ISI- between the prime and the mask was 314 ms. On the remaining half of trials, the 33 ms prime was immediately followed by the pattern mask (prime-mask ISI = 0-ms) that remained on the screen (during 567 ms) until the presentation of the target.

Using similar masking conditions, several studies have reported reliable semantic NP from single ignored primes. More specifically, it appears when the pattern mask is delayed (Daza et al., 2007; Wang et al., 2014, Exp. 1, see also Exp. 5), or when the mask is immediate, but followed by a long blank ISI before the target (Noguera et al., 2007, Exp. 5; Ortells et al., 2003, Exp. 4; Wang et al., 2014, Exps 3 and 4; Wang et al., 2018). In clear contrast, NP is systematically eliminated (or even reversed to PP) when the ignored prime word is immediately followed by a persistent mask that remains on the screen throughout the prime-target ISI interval (Daza et al., 2007; Wang et al., 2014, 2018).

By assuming that NP reflects the involvement of controlled processes, one could argue that presenting a persisting masking pattern immediately following the prime offset would impede conscious processing of the prime, thus reducing NP. Yet, it is not clear if conscious awareness is a necessary condition for NP in the single word paradigm, as recent studies have reported reliable semantic NP even when the prime is below an objective threshold of awareness (Milliken et al., 1998; Frings and Wentura, 2005; Wang et al., 2018, Exp. 2; Neill and Kahan, 1999).

A more plausible hypothesis to explain the lack of NP with an immediate (and persistent) mask is the one put forward by Wang et al. (2014, 2018). Based on an idea originally developed by Houghton et al. (1996), Wang et al. (2014, 2018) suggested that a masking pattern that persists at the same position where the upcoming target will appear, could generate a continuous perceptual input that would interfere with the buildup of the top-down inhibition resulting from an ignore instruction. That interference process would explain the lack of NP under an immediate masking condition. By contrast, when there is an ISI interval between the prime and the mask, as occurs for example with a delayed masking condition, the masking stimulus would not interfere with the implementation of the inhibition, so an ignore instruction could lead to reliable NP.

A second goal of the present research was to investigate whether the differential priming pattern under immediate vs. delayed masking conditions observed by some previous studies (Daza et al., 2007; Wang et al., 2014) could be modulated by WMC. If a persisting immediate mask interferes with attentional inhibition, then we should not expect to find differences between high vs. low WMC participants when the ignored prime is immediately followed by a persisting mask. Under such immediate masking condition, both groups of participants should show a similar pattern, namely a lack of NP (or even an opposite PP effect). In clear contrast, the ignored single prime followed by a delayed mask could produce reliable semantic NP only for participants with high WMC, but not for those with low WMC (i.e., a three-way interaction between Masking Type, Relatedness and WMC).

## Materials and Methods

### Participants Screening for WMC and Attention Control

A sample of 219 native Spanish speakers (mean age = 24.5 years, range 17–53, SD = 8.8) was tested for WMC. All of them had normal or corrected to normal vision.

Previous research on the relationship between WM and Attention sometimes pre-tested the sample using a battery of tests to measure attentional control. These measures include some WM tasks (e.g., OSpan) as well as other tests of inhibitory control such as the Antisaccade task or Stroop (Hutchison, 2007; Hutchison et al., 2014; Foster et al., 2015; Ortells et al., 2016a; Robison and Unsworth, 2018; Noguera et al., 2019). These attentional control measures have been used to test their correlation with WMC scores and thus provide further validation of the methods used (Hutchison, 2007; Hutchison et al., 2014; Ortells et al., 2016b). In addition, they have also demonstrated consistent age-related differences in WMC (De Jong, 2001; Noguera et al., 2019). Consequently, participants performed the Spanish adaptations of the automated versions of the Operation and Symmetry Complex Span tasks (Unsworth et al., 2005, 2009; Ortells et al., 2016a) as well as versions of the Antisaccade and the Stroop tasks (Hutchison, 2007; Kane et al., 2001; Ortells et al., 2016b). The presentation order of the tasks was counterbalanced across participants.

In the automated Operation Span task (AOSPAN; Unsworth et al., 2005) participants are required to solve simple arithmetic operations while they retain a variable set of letters respecting the order in which they were displayed. The number of operation-letter pairs per series varied from three to seven (with three series of each length), and participants were told that both arithmetic operations and letter recall tasks were equally important. The dependent measure computed for each participant was the sum of letters correctly recalled from set that were recalled without intrusions (G*lobal Aospan Score*), with the total score ranging from 0 to 75.

In the Automated Symmetry Span task (ASYMSPAN; Unsworth et al., 2009) participants have to recall variable sequences of red squares in the same order they are displayed within a 4 × 4 matrix of blank squares, while performing a vertical symmetry-judgment task on an 8 × 8 geometric figure of black and white squares. The number of symmetry figures-square locations per series varied from two to five (with three series of each length) for a total of 42 trials on the task. The total score for each participant (*Global Asymspan Score*) had a maximum of 42, reflecting the number of locations recalled in the correct serial position without intrusions.

A *z*-score WMC composite was also calculated by averaging across the two complex span tasks z-scores for each participant. We then computed quartiles for our 219 participants with *z*-scores of −0.53 and +0.57, which corresponded to the lower and upper quartiles, respectively.

In the Antisaccade task, participants had to identify a letter (*O* or *Q*), that is briefly presented (100 ms) followed by a mask. This target letter could either appear on the same (prosaccade) or on the opposite (antisaccade) visual field of an asterisk that appeared 300-ms before the target. The asterisk location varied randomly from trial to trial to stop participants from guessing the location. The order of antisaccade and prosaccade blocks was counterbalanced across participants. Participants were encouraged to move their eyes to the location of the asterisk in the prosaccade block to make easier the detection of the target letter. By contrast, in the antisaccade block, they had to look away from the asterisk to identify the target on the opposite visual field before it disappeared (Ortells et al., 2016a; further discussion in Noguera et al., 2019).

The orienting of attention to the asterisk in the prosaccade block is more automatic and less dependent on executive control than in the antisaccade one. The differential performance between the prosaccade and antisaccade blocks provided an additional index of attentional control. If individuals with a lower WMC have mainly a general decline in processing speed, then their responses should be slower than those from high-WMC individuals in both the antisaccade and prosaccade trials. But if low-WMC participants present a decreased attentional control capacity, then their performance could be much worse on the antisaccade than on prosaccade trials.

In the Stroop task, participants had to respond to the ink color (red, green, or blue) of a central word (RED, GREEN, or BLUE) that stays on the screen until response. All participants completed a practice block of 24 trials followed by an experimental block with 60 trials, from which 42 trials (70%) were congruent and 18 trials (30%) incongruent. Research has found that differences in Stroop interference between individuals with high vs. low WMC emerge mainly when there is a relatively low number of incongruent trials and/or congruent items are included in the stimulus list (Kane and Engle, 2003; Hutchison, 2007). These task conditions are indeed more sensitive to individual differences as they place greater demand on working memory. The low frequency of incongruent trials makes it harder to stay focused on the color naming task and to avoid reading the word (Kane and Engle, 2003), and low WMC individuals seem to be specially affected by this.

### Participants

Thirty-two high (23 females) and thirty-two low (24 females) WMC participants, who had, respectively WMC composite z-scores falling within the upper (>+0.57) and lower (<−0.53) quartiles of our 219-participants pool (see Table 1 below), were selected for the NP study. These sample sizes were similar or even greater than those used by previous studies addressing semantic NP from single primes (Daza et al., 2007, *n* = 24; Wang et al., 2014, *n* = 25; Wang et al., 2018, *n* = 24, Experiments 2–4), and studies investigating the dependence of NP on either aging (Mayas et al., 2012, *n* = 18), WM load (Chao and Yeh, 2008, *n* = 20; De Fockert et al., 2010; *n* = 20) or individual differences in WMC (Conway et al., 1999, *n* = 23–26; Ortells et al., 2016b, *n* = 24). We further performed a *post hoc* power analysis using G^∗^Power software 3.1.9.2 (Faul et al., 2007) to determine the power of both main and interaction effects (repeated measures) in our study. With an alpha = 0.05, a medium effect size (*d* = 0.36) and total sample size = 64, the analysis revealed statistical power greater than 0.99. The minimum statistical power of correlations was 0.91. Participants were between 18 and 48 years old (*M* = 25.38, *SD* = 9.15 for the high WMC group; *M* = 24.78, *SD* = 8.65 for the low-WMC group). A signed written consent was obtained from all participants, with the study being approved by the University of Almería Human Research Ethics Committee and conducted in accordance with the Declaration of Helsinki.

**TABLE 1:** Summary statistics for performance in the complex span WM tasks (Aospan, Asymspan, and *z*-score global composite) and attentional control tasks (Antisaccade and Stroop congruency) by Low-WMC and High-WMC groups.

	Low-WMC Mean (SD)	High-WMC Mean (SD)	Group differences	Effect Size *d*
Span WM tasks				
Aospan score	16.9 (6.3)	50.56 (6.2)	*t*(62) = 21.5	5.37
Asymspan score	9.8 (6.02)	24.7 (4.8)	*t*(62) = 10.9	2.74
z-score composite	−0.99 (0.45)	0.987 (0.31)	*t*(56)^a^ = 20.4	5.06
Antisaccade task				
Prosaccade condition				
RT (ms)	517 (22.15)	438 (15.5)	*t*(55)^a^ = 2.90	4.13
ACC (%)	94 (8)	98 (3)	*t*(40)^a^ = 2.34	0.66
Antisaccade condition				
RT (ms)	699 (25)	546 (23)	*t*(62) = 4.48	6.4
ACC (%)	74 (2)	94 (1)	*t*(45)^a^ = 8.67	12.6
Antisaccade Differences (prosaccade−antisaccade)				
RT (ms)	−181.9 (112.2)	−108.1 (86.6)	*t*(62) = 2.95	0.74
ACC (%)	0.20 (0.11)	0.036 (0.06)	*t*(45)^a^ = 7.39	1.85
Stroop task				
Congruent condition				
RT (ms)	653 (29.5)	638 (29.4)	*t*(62) = 0.36	0.50
ACC (%)	99 (1)	99 (1)	*t*(62) = 0.25	0
Incongruent condition				
RT (ms)	780 (35.5)	715 (31.2)	*t*(62) = 1.38	1.96
ACC (%)	96 (6)	99 (2)	*t*(38)^a^ = 2.60	0.7
Stroop Congruency (congruent−incongruent)				
RT (ms)	−126.9 (103.8)	−76.8 (70.4)	*t*(62) = 2.26	0.56
ACC (%)	0.034 (0.06)	0.001 (0.02)	*t*(39)^a^ = 2.97	0.54

*All *p* values < 0.05. ^a^ Correction of *df*s for unequal variances. The possible range of scores for Operation span and for Symmetry span tasks are 0–75, and 0–42, respectively.*

### Stimuli and Apparatus

The stimulus set was similar to that recently used by Ortells et al. (2016a). It consisted of 32 familiar Spanish nouns of 4–6 letters length (16 animals and 16 body-parts) selected from the intra-categorical associative norms published by Callejas et al. (2003). 16 of these words (8 from each category) appeared only as primes and 16 words were used exclusively as targets. Half of the prime and target words from each category were randomly chosen to appear in the immediate mask trials, while the rest appeared in the delayed condition. The assignment of each word set to the masking condition was counterbalanced across participants. For each participant and masking condition, the same prime and target words (four pairs from each semantic category) were presented on both related and unrelated trials. The related pairs were highly associated and members of the same category (i.e., the first ranked exemplar on both forward and backward directions, such as LION-tiger or THIGH-leg, Callejas et al., 2003). The unrelated word pairs were created by re-pairing the former prime and related target words in a pseudorandom way, such that the prime words from each semantic category were followed by low associated target words belonging to the other semantic category (e.g., LION-leg; THIGH-tiger).

**FIGURE 1:**
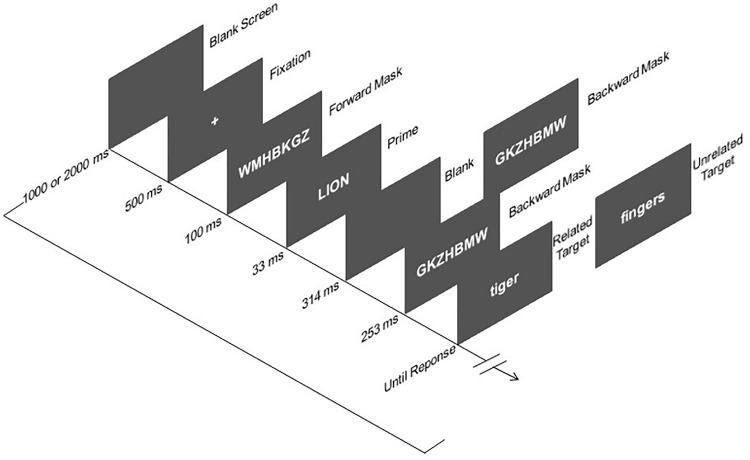
Sequence and time of events in Experiment. The word stimuli shown here for related and unrelated trials have been translated from Spanish to English.

Stimulus presentation and response recordings were controlled by E-prime software (Psychology Software Tools Inc)^1^. All stimuli were presented on the center computer screen at a viewing distance of approximately 60 cm. Each trial consisted of a sequence of the following displays (see Figure 1): Blank screen, fixation, forward mask, prime, either backward mask or blank screen plus backward mask (depending on masking condition), and target. The fixation display consisted of a central white cross (+) presented on a black background. The forward and backward masks were composed of random strings of seven white uppercase consonants at the center of the screen (e.g., WMHBKGZ), subtending a visual angle of about 2.46° wide and 0.49° high. The prime and probe displays contained a single word presented at the center of the screen in uppercase and lowercase, respectively. Both the prime and target words subtended an averaged visual angle of 2.21° wide and 0.49° high. The target word presented on every related or unrelated trial never shared the first (or last) letter or syllable with the preceding prime word, in order to avoid the orthographic overlapping between prime and target stimuli (Callejas et al., 2003; Ortells et al., 2016b).

### Design and Procedure

Instructions for completing the task were both presented on the screen and orally described. The sequence of the events were: (1) Blank screen presented for a variable duration (1000 vs. 2000 ms); (2) Fixation (+) presented for 500 ms; (3) Forward mask (random string of consonants) at the center of the screen for 100 ms^2^; (4) Prime display, to be ignored by the participants, containing a single uppercase word centrally presented for 33 ms; (5) either a backward mask (a different random string of consonants) presented for 567 ms until the target onset (immediate masking condition), or a 314-ms blank screen followed by a 253-ms mask (delayed masking condition), and then for the target (thus resulting in a fixed prime-target SOA of 600 ms), with the immediate and delayed masking conditions varying randomly from trial to trial; (6) Target display consisting of a lowercase word centrally presented until response, on which participants made a categorization judgment (animal vs. body part). Participants were required to respond as fast and as accurately as possible to the target category by pressing either the “c” or “m” key on the computer keyboard, with the mapping between categories and response keys being counterbalanced across participants. They were also encouraged to consider the preceding prime word as a distractor that they should actively ignore on every trial.

Participants performed the task in a single experimental session (lasting about 35 min) of 16 practice trials and 256 experimental trials, which consisted of 4 consecutive blocks of 64 trials each. Within each block of 64 trials, there were 32 immediate, and 32 delayed masking trials, which varied randomly within the block, with different prime-target pairs within each masking condition (words in the immediate mask condition for half of participants appeared in the delayed condition for the remaining half, and vice versa). Within each masking 32 trial-set, there were 16 related and 16 unrelated trials. Within each 64-trials block, each word (prime and target) appeared four times, twice followed by a semantically related target from the same category and twice by an unrelated word target from the opposite category.

The WMC was manipulated as the between-subject factor at two levels (High vs. Low WMC); and Prime-Target Relatedness (Related vs. Unrelated), and Masking Type (Delayed vs. Immediate mask) were manipulated as the within-subject factors, with a different random order for each individual. Half of the trials were “Related” and half were “Unrelated.” Within each of these conditions, the immediate and delayed masking trials occurred equally often and in a randomized order.

After completing the categorization task, participants performed a prime visibility test to assess their awareness about the prime words followed by both immediate and delayed masking patterns. This test included 8 practice trials followed by 64 experimental trials, 32 trials for each masking condition. The sequence and timing of events were identical to those of the categorization task, with the difference that participants were now instructed to categorize the prime, rather than the target stimulus. They were told that the prime word could be either an animal or a body-part with an identical probability (0.50). If they were unable to categorize the prime, then they were encouraged to make the best guess without time limit.

## Results

### Working Memory (Complex Span) and Attention Control (Antisaccade) Tasks

Table 1 shows descriptive statistics (means and standard deviations) for performance by Low- and High-WMC participants in the two complex span WM tasks (global span and *z*-composite scores), and the two attentional control tasks (Antisaccade and Stroop). As explained earlier, participants were assigned to each WMC group on the basis of their compound global *z*-score in the two span WM tasks with no overlap. Independent samples *t*-tests also found improved performance of the high WMC group compared to the Low WMC one for each separate WM task (see Table 1). The high WMC groups was generally faster and more accurate than the low WMC one in each of these two attention control tasks. More importantly, the high WMC group also demonstrated better control than the low WMC one in both the Antisaccade task (with smaller differences between prosaccade and antisaccade conditions), and the Stroop task (with smaller differences between congruent and incongruent trials). These results support the idea that lower WMC seems to be associated with not only a slower processing speed or task performance, but also with a decreased capacity for attentional control (see also Noguera et al., 2019 for similar pattern of performance when comparing younger vs. older adults).

Additional mixed analyses of variance (ANOVAs) supported these impressions. WMC (high vs. low) was included as a between-participants factor, and either Saccade Type (antisaccade vs. prosaccade), or Stroop Congruency (congruent vs. incongruent), as the within-participants variable. Results from the Antisaccade task showed significant main effects for both WMC group [ACC: *F*(1,62) = 52.3, *p* < 0.001, η^2^ = 0.46; RTs: *F*(1,62) = 17.05, *p* < 0.001, η^2^ = 0.22] and Saccade Type [ACC: *F*(1,62) = 112, *p* < 0.001, η^2^ = 0.64; RTs: *F*(1,62) = 133.9, *p* < 0.001, η^2^ = 0.68], such that performance was reliable better for participants with a higher WMC, and for prosaccade, relative to the antisaccade trial block. More importantly, the there was also a reliable interaction between WMC and Saccade Type in both accuracy [*F*(1,62) = 54.6, *p* < 0.001, η^2^ = 0.47], and response latency [*F*(1,62) = 8.71, *p* = 0.04, η^2^ = 0.12]. This interaction revealed that the improved performance observed in the high WMC group, compared to the low one, was greater in the antisaccade condition than in the less attention demanding prosaccade condition (see Table 1).

A fairly similar pattern was found in the Stroop task. There was again a reliable main effect for WMC group [ACC: *F*(1,62) = 4.7, *p* = 0.03, η^2^ = 0.07; RTs: *F* < 1], which revealed a better performance for the high-WMC relative to the Low-WMC group. The main effect of Stroop Congruency was also significant [ACC: *F*(1,62) = 8.2, *p* = 0.06, η^2^ = 0.12; RTs: *F*(1,62) = 84.4, *p* < 0.001, η^2^ = 0.58], such that performance in the Stroop task was better on congruent than on incongruent trials. More importantly, there also was significant the interaction between WMC and Stroop congruency [ACC: *F*(1,62) = 8.81, *p* = 0.004, η^2^ = 0.125; RTs: *F*(1,62) = 5.11, *p* = 0.027, η^2^ = 0.07]. Thus, the differences in performance between high and low-WMC participants were much greater in the conflicting (incongruent) trials, than in the non-conflict (congruent) trials (see Table 1)^3^.

### Priming Task

Trials containing an incorrect response (3.56% of total) or those with RTs falling more than 2.5 standard deviations from the overall mean RT (2.7% of trials) were removed from analyses. Mean RTs and percentages errors per participant and per condition were included in two separate analyses of variance (ANOVAs) with WMC (High- vs. Low-WMC) as a between-participants factor, and Masking Condition (Delayed vs. Immediate), and Prime-target Relatedness (Related vs. Unrelated) as within-subjects variables. Mean RTs and mean error percentages as a function of Masking condition and Relatedness for each WMC group are shown in Table 2.

**TABLE 2:** Mean (SD) reaction times (in milliseconds), and error percentages (in %) as a function of working memory capacity (Low vs. High WMC), Prime-Target Relatedness (Related vs. Unrelated) and Masking Type (Delayed vs. Immediate Mask).

	WM Capacity	
	Low-WMC	High-WMC
Delayed Mask		
Related	696 (160.3)	673 (118.5)
	3.5 (0.04)	4.00 (0.04)
Unrelated	714 (192.7)	647 (108.4)
	3.0 (0.04)	2.8 (0.03)
Immediate Mask		
Related	694 (146.1)	650 (110.1)
	4.5 (0.05)	3.3 (0.03)
Unrelated	702 (148.7)	648 (104.3)
	3.8 (0.03)	3.6 (0.03)

The analysis of error rates showed no reliable effect (all *p* values >0.09). In the analysis of RTs, there was a reliable interaction between WMC and Relatedness [*F*(1,62) = 7.86, *p* = 0.007, η^2^ = 0.113], such that the ignored prime words produced a reliable semantic NP effect in participants with a high WMC [−14 ms; *F*(1,31) = 5.24, *p* = 0.029, η^2^ = 0.15), whereas an opposite (thought non-significant) positive priming (PP) was found for low-WMC participants [+13 ms; *F*(1,31) = 3.08, *p* = 0.089, η^2^ = 0.09). More interesting yet, there also was reliable the three-way interaction between WMC, Masking condition, and Relatedness [*F*(1,62) = 5.46, *p* = 0.023, η^2^ = 0.081].

**FIGURE 2:**
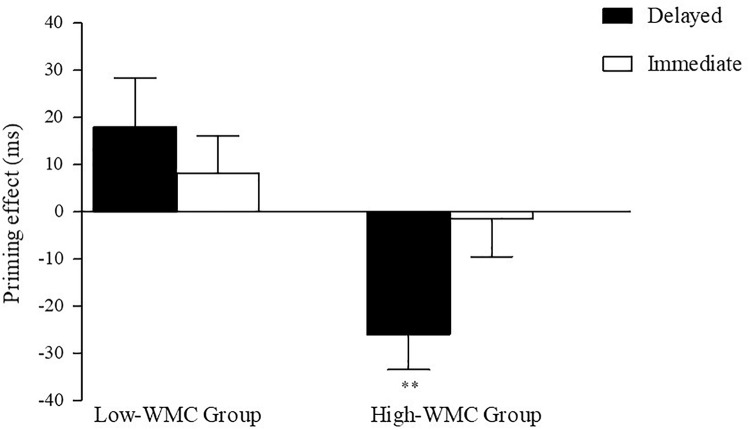
Semantic priming effects (Unrelated–Related) for Delayed and Immediate Masking conditions for Low-WMC and High-WMC participants. The standard error of priming scores for each condition is depicted using vertical lines. Statistically significant differences are highlighted by asterisks (**p < 0.01).

**FIGURE 3:**
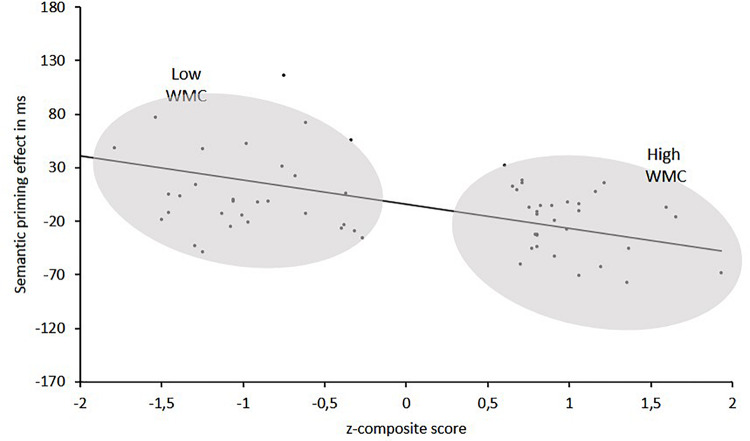
Semantic priming effects (in ms) as a function of *z*-composite scores in WM span tasks for High-WMC and Low-WMC groups.

The interaction was followed up by two separate ANOVAs for each WMC group. The results of these analyses can be summarized as follow (see Figure 2): The High-WMC group showed a reliable main effect for Relatedness (Related = 661 ms; Unrelated = 647 ms; *F* (1, 31) = 5.24, *p* = 0.029, η^2^ = 0.15). However, this NP effect reached significance only with the delayed mask (−26 ms; *p* = 0.001), not with the immediate (persistent) mask (+2 ms), as qualified by a significant interaction between Masking type and Relatedness [*F*(1,31) = 6.46, *p* = 0.0016, η^2^ = 0.17]. In clear contrast, the Low-WMC group only showed a non-significant tendency for facilitatory priming (+13 ms), which was not modulated by the masking type (Delayed mask = +17 ms; Immediate mask = +10). Further evidence that obtaining semantic NP under delayed masking in our task depends critically on WMC is depicted in Figure 3, which shows the relationship between WMC and NP within each group. The present results are thus consistent with previous reports suggesting that a differential availability of control (WM) resources reliably modulates NP (Engle et al., 1995; Chao and Yeh, 2008; Ahmed and De Fockert, 2012; Ortells et al., 2016a).

#### Prime Visibility Test

Prime visibility was assessed under both the immediate and delayed masking conditions, with the signal detection measure *d’* being obtained with each masking type for each participant. The measures were achieved, considering one level of the prime category (e.g., animal) as signal and the other level (e.g., body part) as noise (for a similar procedure see Ortells et al., 2013; Ortells et al., 2016b; Wang et al., 2018). Discrimination for primes on delayed masking trials (*d’* = 0.12) was significantly greater [*t*(63) = 2.13, *p* = 0.037] than that on the immediate masking ones (*d’* = 0.02). These differences in discrimination for the two masking conditions remained fairly stable across the two WMC groups (interaction discrimination by group *F* < 1). In addition, *d’* with the delayed mask was clearly above zero [*t*(63) = 4.42, *p* = 0.001], but for the immediate mask did not deviate from zero (*t* < 1). These findings suggest that the primes were below the objective consciousness threshold with the immediate mask, and above it with the delayed one.

Lastly, there was a lack of correlation observed between the *d’* values for each participant and its corresponding priming scores in either group or type of mask (Immediate, High-WMC: *r* = 0.07, *p* > 0.68; Low-WMC: *r* = 0.16, *p* > 0.33; Delayed, High-WMC: *r* = −0.20, *p* > 0.27; Low-WMC: *r* = 0.13, *p* > 0.49). The absence of a correlation between these two indices of prime processing, a direct and an indirect one, is common in the literature (Ortells et al., 2013, 2016a; Wang et al., 2014, 2018) and support the idea that they map on to different processes.

## General Discussion

There now is ample evidence showing a dependence of NP on cognitive control (working memory) resources. Thus, the probability that an ignored (or selected against) prime stimulus gives rise to a reliable NP effect in a selective attention task is significantly reduced in older adults (relative to younger participants), in individuals with a lower WMC, or when participants are required to perform a concurrent task demanding a high memory load (Engle et al., 1995; Chao and Yeh, 2008; De Fockert et al., 2010; Chao, 2011).

Note that most of these studies used a standard NP procedure, in which (a) the ignored prime is presented as the target stimulus on the following probe display, and (b) the to-be-responded target is presented among competing distractors on both the prime and probe displays. But in a recent study using a NP task in which the prime (and probe) display contained a single word stimulus in an otherwise empty field, Ortells et al. (2016b) reported that individual differences in WMC could also modulate NP even at a semantic level of representation.

Semantic NP has been largely considered a weak and difficult-to-replicate effect compared to repetition NP (Fox, 1995; Macleod et al., 2002). However, further research has convincingly demonstrated that semantic NP consistently appears when several boundary conditions are met (Daza et al., 2007; Noguera et al., 2007; Wang et al., 2014, 2018): (1) instructing participants to actively ignore the prime stimulus; (2) presenting prime-target pairs that are highly associated (e.g., *dog-cat*); (3) using a prime-probe SOA interval that allows an efficient implementation of controlled processes (i.e., 600 ms or longer); and (4) using a demanding forced-choice task on the probe (e.g., lexical decision, semantic categorization. All these conditions were included in Ortells et al. (2016b) which participants were also distributed according to their high vs. low WMC. Their results showed that, when instructions encouraged attending to the single prime word, a similar size of facilitatory priming effect was found for both high- and low-WMC individuals. In clear contrast, when participants had to ignore the single prime, a reliable NP effect appeared in the high-WMC group, with the low-WMC group exhibiting a positive priming effect, demonstrating that semantic NP strongly depends on WMC.

Thus, the individual differences in the availability of WM cognitive resources, in addition to the boundary conditions mentioned above, should be on the list of determinants of semantic NP. The lack of assessment of WMC in traditional semantic NP studies could be relevant to explain the volatility of the effect.

It should be noted that in Ortells et al. (2016a) study participants were required to continuously change their mental-set about how to process the single prime, as attentional instructions to either attend to or ignore the prime changed randomly from trial to trial, increasing even more an already demanding task. Thus, we set as a first aim to find out whether a similar dependence of semantic NP on WM resources could be observed with a NP task that did not require task switching. Accordingly, our participants were encouraged to continuously ignore (but not attend to) the single prime on every trial, an instruction condition which resembles the semantic NP task previously used by Daza et al. (2007).

The results of the present research replicate and extend those obtained by Ortells et al. (2016b) in demonstrating that an ignored prime word produced reliable semantic NP but only in the higher-WMC, not in the lower-WMC group. These are consistent with inhibitory accounts of NP, which assume that attention inhibition reflects a resource demanding (controlled) processes. Thus, a high-WMC could involve a greater ability of attention control mechanisms to efficiently inhibit the processing of the ignored prime. This could explain why only the higher-WMC, but not the lower-WMC group showed reliable NP from ignored primes.

Further evidence in support of the inhibitory hypothesis comes from the relationship observed between performance in the two WM span tasks (as reflected in *z*-composite scores, whether we used a global or partial scoring method; see Footnote 2), and their performance in both the Antisaccade and Stroop congruency tasks. Relative to low-WMC participants, individuals with a higher-WMC were faster and more accurate in both attention control tasks (see Table 1). Even more relevant, they also showed a better control on both the Antisaccade (differences between prosaccade and antisaccade conditions), and the Stroop (differences between congruent and incongruent conditions) tasks. Thus, regardless whether we treated WMC as a grouping factor or as a covariate, the results clearly show a modulation of performance in these attention control tasks by their WMC. Overall, these findings clearly support that greater availability of WM resources results in increased inhibitory control.

There is some recent evidence suggesting that the ability to implement attention inhibitory processes could be slowed rather than impaired in older adults or in younger individuals with a lower-WMC (Gazzaley et al., 2008). For example, inhibition of return seems to be delayed in older adults compared to younger individuals (Li et al., 2020). By using different strategic priming tasks, Noguera et al. (2019) have recently demonstrated that the ability to efficiently implement expectancy-based facilitatory strategies would also slowdown in normal aging. Based on these findings, it is possible that relative to High-WMC individuals, those with a lower-WMC need more time to develop a strategic response. If so, it might be possible that an ignored prime can result in reliable semantic NP in Low-WMC participants using SOAs longer than the ones used here. This could be an interesting issue for future research.

On the other hand, unlike the study by Ortells et al. (2016a) the to-be-ignored prime in the present research could be either clearly visible (delayed mask condition), or not (immediate mask condition). There are several previous consistent demonstrations that the NP effect from single ignored prime words is systematically eliminated when the prime offset is immediately followed by a persisting pattern mask that remains on the screen either for a relatively long duration, or during all the prime-target ISI interval (Daza et al., 2007; Wang et al., 2014, 2018). Accordingly, our second goal was to determine whether the differential priming pattern as a function of masking condition that has been previously reported, could also be sensitive to individual differences in WMC. In support of that hypothesis, we found a reliable three-way interaction between prime-target Relatedness, Masking condition, and WMC. As expected, both participants with high- and those with low-WMC showed a very similar task performance when a persisting pattern mask immediately followed the ignored prime. Namely, no reliable NP was found for any WMC group. In stark contrast, when the mask onset was delayed, the ignored prime produced reliable NP but only for high-WMC participants, with low-WMC individuals showing an opposite (thought non-significant) facilitatory priming.

It has been suggested that presenting a persistent pattern mask immediately following the prime offset, would impede the conscious (controlled) processing of that stimulus, thus explaining the removal of NP under such masking condition (Daza et al., 2007). Our results from the prime visibility test showed in fact that participants from both WMC groups could discriminate the prime stimulus clearly above the objective threshold for conscious awareness under the delayed, but not under the immediate masking condition. Note however, that the role of prime awareness in single NP remains a debated issue, and there are some recent reports of reliable single NP even when the to-be-ignored prime is subliminally presented (Milliken et al., 1998; Neill and Kahan, 1999; Wang et al., 2018).

A perhaps more plausible account of the dependence of single NP on the absence vs. presence of a persisting mask has recently been suggested by Houghton et al. (1996), Wang et al. (2014, 2018). According to these authors, presenting a persisting pattern mask immediately following an ignored prime would create a perceptual input that would interfere with the buildup of attention inhibition, thus explaining the elimination of NP with an immediate mask. But the implementation of inhibition would be possible when there is an ISI interval long enough between the prime and the mask (or between a mask and the upcoming target). Our findings would be consistent with that hypothesis. Assuming that the use of a persisting immediate mask would interrupt the buildup of attention inhibition, we did not expect to find differences between low-WMC and high-WMC groups under such masking condition. In fact, we found a consistent relation between WMC scores and the ignored priming effects only with a delayed mask (see Figure 3).

The findings that an ignored prime followed by a delayed mask gives rise to reliable NP in participants with higher-WMC, but not in those with lower-WMC, could be well accommodated by both the inhibition (Hasher et al., 1999, 2007) and the executive attention (Engle and Kane, 2004) theories of working memory. Thus, a high-WMC could reflect an improved ability either to inhibit or suppress task-irrelevant information (e.g., an ignored prime), or to maintain in an active state the task-relevant information while potentially competing irrelevant distractors are blocked. Whether individual differences in WMC could modulate not only behavioral measures, but also electrophysiological (ERP) correlates of semantic NP remains an interesting matter for future research.

## Data Availability Statement

The datasets generated for this study are available on request to the corresponding author.

## Ethics Statement

The study was reviewed and approved by the University of Almería Human Research Ethics Committee, and was conducted in accordance with the Declaration of Helsinki. The participants provided their written informed consent to participate in this study.

## Author Contributions

MM and JO developed the concept and the design of the experimental work and were responsible for writing the manuscript. MM, JO, CN, and IC actively participated in the implementation of the experimental tasks, data collection, and data analyses. All authors supervised the processes of accomplishing the study, substantially contributed to the interpretation of data, to writing and reviewing the manuscript, as well as to approving the final version of the manuscript.

## Conflict of Interest

The authors declare that the research was conducted in the absence of any commercial or financial relationships that could be construed as a potential conflict of interest.

## Footnotes

1 www.pstnet.com/eprime

2 Given that some recent work has reported reliable semantic NP from an ignored single prime even when its presentation was completely subliminal (Wang et al., 2018) in our NP task we also presented a forward mask that preceded (100 ms before) the prime word onset. The inclusion of a premask in our study was aimed to increase the likelihood that an ignored prime preceded and immediately followed by a persistent mask could be below both “subjective” and “objective” thresholds for awareness (*a d’* at chance in a further prime visibility test; see also Ortells et al., 2016a).

3 In order to further examine whether the differential performance by participants in the different (contrasting) conditions of the two attention control tasks was modulated by individual differences in WMC, we conducted a series of ANCOVAs in which WM capacity (z-composite global score of each participant) was treated as a continuous covariate (for similar analyses, see Hutchison, 2007; Richmond et al., 2015; Maldonado et al., 2018; Ortells et al., 2018). The results showed that the main effects of Saccade condition and Stroop congruency were qualified by reliable interaction effects between WMC and both Saccade type [ACC: *F*(1,62) = 41.3, *p* < 0.001, η^2^ = 0.40; RTs: *F*(1,62) = 8.4, *p* = 0.005, η^2^ = 0.12], and Stroop interference [ACC: *F*(1,62) = 9.5, *p* = 0.003, η^2^ = 0.13; RTs: *F*(1,62) = 3.9, *p* = 0.05, η^2^ = 0.08]. The same ANCOVA analyses were also conducted on partial scores instead of global measures of participants’ performance in the WM span tasks (i.e., the sum of items recalled in the correct serial position, regardless of whether an entire trial was recalled correctly; see for example (Redick et al., 2012; Richmond et al., 2015). The results of these ANCOVAs were very similar to those observed with global or absolute span scores. Namely, the main effects of Saccade type and Stroop interference were again qualified by reliable interaction effects between WMC and both Saccade type [ACC: *F*(1,62) = 41.4, *p* < 0.001, η^2^ = 0.40; RTs: *F*(1,62) = 9.8, *p* = 0.003, η^2^ = 0.14], and Stroop congruency [ACC: *F*(1,62) = 9.7, *p* = 0.003, η^2^ = 0.13; RTs: *F*(1,62) = 4.7, *p* = 0.03, η^2^ = 0.07].
